# Integrated Approach of Hematological Parameters and Glutathione as Predictors of Pulmonary TB Evolution: A Comprehensive Review

**DOI:** 10.3390/jcm15031017

**Published:** 2026-01-27

**Authors:** Ionela Alina Grosu, Mona Elisabeta Dobrin, Corina Marginean, Irina Mihaela Esanu, Oana Elena Melinte, Ioan Emanuel Stavarache, Stefan Dumitrache-Rujinski, Ionel-Bogdan Cioroiu, Radu Adrian Crisan-Dabija, Cristina Vicol, Antigona Carmen Trofor

**Affiliations:** 1 Grigore T. Popa University of Medicine and Pharmacy Iasi, 700115 Iasi, Romania; ionela.grossu@yahoo.com (I.A.G.); irina.esanu@umfiasi.ro (I.M.E.); oana-elena.melinte@umfiasi.ro (O.E.M.); stavarash@yahoo.com (I.E.S.); radu.dabija@umfiasi.ro (R.A.C.-D.); cristina.vicol@umfiasi.ro (C.V.); antigona.trofor@umfiasi.ro (A.C.T.); 2 Biochemistry Department, Clinical Hospital of Pulmonary Diseases, 700116 Iasi, Romania; elisabeta-mona.dobrin@pneumo-iasi.ro; 3 Oncology and Palliative Care Department, George Emil Palade University of Medicine, Pharmacy, Science and Technology of Târgu Mureș, 540139 Târgu Mureș, Romania; 4 Department of Preclinical Disciplines, Faculty of Medicine, “Apollonia” University of Iaşi, 700511 Iasi, Romania; 5 Department of Pneumophtysiology I, “Carol Davila” University of Medicine and Pharmacy, Dionisie Lupu Street, Nr. 37, Sector 2, 020021 Bucharest, Romania; stefan.dumitrache@umfcd.ro; 6 Romanian Academy-Iasi Branch, Research Center for Oenology, 700490 Iasi, Romania; bogdan.cioroiu@acadiasi.ro

**Keywords:** tuberculosis, systemic immune-inflammation index, systemic inflammation response index, prognostic inflammatory index, glutathione

## Abstract

In recent decades, the burden of TB has been gradually declining; however, with the emergence of COVID-19 and ongoing political conflicts, including the war in Ukraine, the proper functioning of healthcare services and TB control programs has been jeopardized. Recently, research has emphasized the importance of hematological parameters associated with inflammation, which can be easily analyzed through routine blood tests. Combining these parameters may have predictive value for various diseases, including pulmonary tuberculosis and even help monitor the effectiveness of treatment. Since there is no single hematological or inflammatory biomarker that provides precise and dynamic information about the success or failure of treatment, identifying individual markers or sets of biomarkers with higher sensitivity and specificity is essential. This is particularly important since sputum culture conversion at two months remains insufficiently sensitive and microscopy conversion has limited sensitivity and specificity in detecting treatment failure. Also, the analysis of the impact of the standard directly observed treatment, short-course regimen on pathogenic mechanisms also focuses on how it influences the interaction between inflammation and oxidative tissue degradation, by measuring plasma levels of glutathione. Utilizing a combination of hematological, inflammatory, and antioxidant biomarkers offers significant insights into systemic inflammatory responses in pulmonary tuberculosis patients, both before commencing treatment and during the entire duration of antituberculosis therapy. Combining different inflammatory parameters into a multiple biomarker can significantly enhance the accuracy of predicting prognosis and response to antibiotic chemotherapy. Identifying an optimal combination of biomarkers with predictive value is crucial for assessing treatment response and evaluating the effectiveness of anti-TB medication. Rather than developing or testing a composite prediction model, this review summarizes reported performance metrics from individual studies and highlights priorities for future prospective validation of integrated biomarker panels.

## 1. Background

Tuberculosis (TB) is a chronic infectious disease caused by *Mycobacterium tuberculosis* (*Mtb*) and remains a major global health threat [1]. Although preventable and treatable, TB is the 13th leading cause of death worldwide and the second deadliest infectious agent after COVID-19 [1,2,3]. The Global Tuberculosis Report 2024 indicates approximately 10.8 million new TB cases globally in 2023, with 1.25 million deaths [3].

A WHO report, developed with the European Centre for Disease Prevention and Control (ECDC) in March 2023, revealed that Romania accounted for 23.8% of 33,520 TB cases across 29 EU countries in 2021—a rate over five times higher per 100,000 inhabitants than the EU average [4]. Romania also reported the highest TB incidence among children under 15, with 9.2 cases per 100,000 children aged 0–4 years and 9.3 cases per 100,000 children aged 5–14 years [4].

Despite progress in TB treatment, some patients remain unresponsive to therapy [3]. While research has yielded more effective drugs and improved regimens, treatment efficacy varies considerably. Antibiotic resistance, poor adherence, and comorbidities can negatively impact outcomes [3,5]. Consequently, patients may develop drug-resistant TB or require complex, prolonged treatment. These challenges underscore the need for personalized approaches. Novel therapeutic strategies and biomarkers enabling early assessment of treatment efficacy are essential to shorten treatment duration, reduce side effects, and improve patient adherence [3,5].

In recent years, several hematological inflammatory indices derived from routine laboratory parameters have emerged as promising biomarkers in TB. These include the systemic immune-inflammation index (SII), systemic inflammation response index (SIRI), and prognostic inflammatory index (PII), all of which are calculated using combinations of neutrophil, lymphocyte, monocyte, platelet counts, and C-reactive protein (CRP). Because these indices rely on routinely available complete blood count and CRP measurements, they are inexpensive, easily accessible, and readily applicable in clinical practice. Biologically, they reflect the balance between innate inflammatory activation (neutrophils, monocytes, platelets) and adaptive immune competence (lymphocytes), which is central to the immunopathogenesis of pulmonary tuberculosis (PTB).

Studies suggest using basic laboratory measurements, such as peripheral blood tests, to predict TB risk [6,7]. Parameters including the SII, SIRI, and PII can aid TB detection and predictability [6,8].

Chronic systemic inflammation in tuberculosis is accompanied by excessive production of reactive oxygen and nitrogen species, leading to a state of sustained oxidative stress that further amplifies tissue damage and immune dysfunction. Within this context, redox homeostasis and antioxidant defense mechanisms become critical determinants of disease progression and treatment response [9,10].

Glutathione (GSH) exerts direct antimycobacterial effects [9] through reductive stress on mycobacteria. Physiological GSH concentrations in macrophages can inhibit *Mtb* growth [9]. GSH is also essential in macrophages, natural killer cells, and T cells, modulating their activation, metabolism, cytokine release, redox balance, and free radical regulation [9,10].

This narrative review aims to synthesize current evidence on the diagnostic and prognostic utility of SII, SIRI, PII, and GSH in PTB, and to propose an integrated conceptual framework for their combined clinical interpretation.

### Immune Modulation by Mtb

TB has evolved alongside humans for millions of years, with current *Mtb* strains tracing back to a common ancestor from 15,000 to 20,000 years ago [11,12]. Evidence of ancient TB infections has been found in Egyptian and pre-Columbian mummies [11,13].

*Mtb* primarily infects the lower respiratory tract and remains a major global cause of death. TB control challenges include poor adherence, drug side effects, and multidrug-resistant strains [14,15]. Approximately 1.7 billion people harbor latent TB infections (LTBI), risking reactivation, especially in immunocompromised individuals [15].

TB spreads through airborne droplets [16]. Inhaled bacilli reach the alveoli and are engulfed by macrophages, neutrophils, and dendritic cells, where they survive by blocking lysosomal destruction [17]. This causes macrophage death and immune cell recruitment, promoting bacterial growth. CD4+ T lymphocyte activation leads to a type IV hypersensitivity response [18].

Following primary infection, outcomes include full recovery, latent infection, or progression to active TB, typically within five years [19]. While most remain asymptomatic, 5–15% of latent cases reactivate [20]. High-risk individuals, including HIV-positive patients and recent contacts, should receive preventive therapy [21]. Active TB can affect the lungs or spread to other organs [22].

As a facultative intracellular pathogen, *Mtb* binds to macrophages and proliferates intracellularly [23,24]. Though alveolar macrophages are the primary entry route, epithelial cells and mucosa-associated lymphoid tissue may also serve as entry points [25,26]. *Mtb* manipulates host signaling and detoxifies reactive oxygen species to enhance survival [27,28,29]. Once inside the lungs, *Mtb* can disseminate via the bloodstream, leading to latent infection if controlled, or active disease if it spreads [18,29,30]. Understanding these mechanisms is vital for developing new therapies and vaccines [30,31].

TB characteristically produces granulomatous lung lesions comprising monocytic infiltrates encircled by CD4+ lymphocytes that localize the infection [32,33]. However, *Mtb* survives within macrophages by inhibiting phagosome–lysosome fusion through cell wall components like lipoarabinomannan and phosphatidylinositol mannoside, evading degradation while maintaining dormancy [32,33].

*Mtb* employs sophisticated immune evasion strategies. Its secretory proteins modulate immune responses through epigenetic reprogramming, inhibition of phagosomal maturation, and cytokine modulation [34]. *Mtb* interacts with Toll-like receptors, initially stimulating protective responses but ultimately causing immunosuppression [35]. The pathogen manipulates its phagosomal environment, engages pattern recognition receptors selectively, and alters T-cell responses [36]. Additionally, *Mtb* hijacks host cell signaling cascades to subvert microbial survival mechanisms [37]. These strategies enable *Mtb* to establish latent or progressive infection despite a functioning immune system.

These immunomodulatory mechanisms directly contribute to the systemic inflammatory and immune dysregulation reflected by hematological indices such as SII, SIRI, and PII, thereby providing a biological basis for their clinical utility as biomarkers of disease activity and prognosis.

## 2. Material and Methods

**Figure 1:**
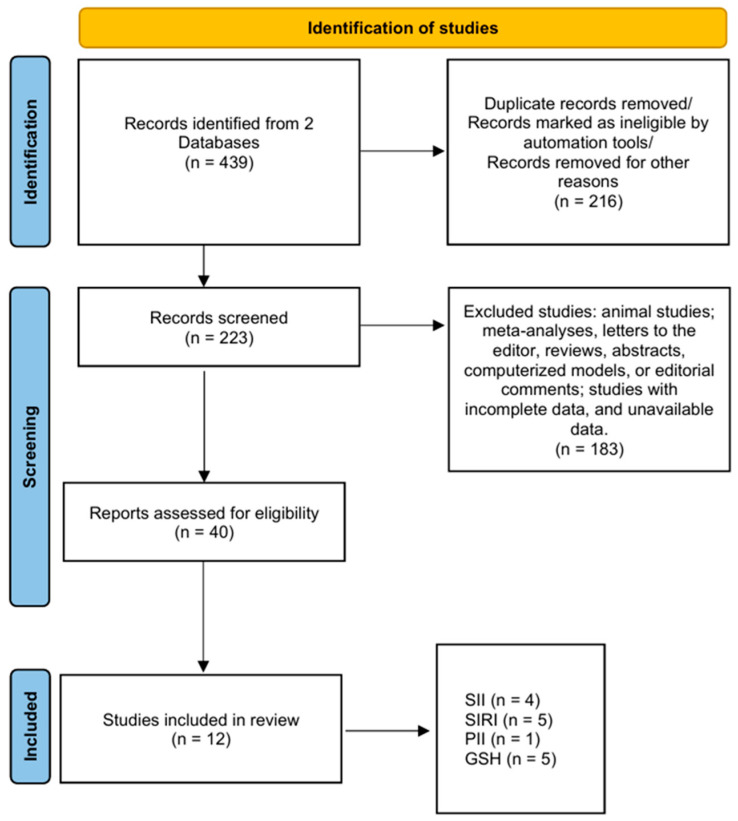
PRISMA diagram depicting the study selection process.

We performed a comprehensive literature review using two principal databases: PubMed and Google Scholar, using the following keyword combinations to meet the aim of this study: “SII” OR “systemic immune-inflammation index” AND “pulmonary tuberculosis”; “SIRI” OR “systemic inflammation response index” AND “pulmonary tuberculosis”; “PII” OR “prognostic inflammatory index” AND “pulmonary tuberculosis”; “Glutathione” OR “GSH” AND “Pulmonary Tuberculosis” OR “Tuberculosis” OR “TB”, and also combinations of these terms to ensure a thorough exploration of the association between the four parameters. We included clinical trials and randomized controlled trials written in English and published from January 2005 up to July 2025. Only original research studies with quantitative methods were selected. Articles on unrelated topics were excluded. A flow diagram of article selection is illustrated in Figure 1. A total of 439 studies were initially identified through database searches. After title and abstract screening, 223 full-text articles were assessed for eligibility. Of these, 12 studies met all inclusion criteria and were included in the final review. Studies were eligible for inclusion if they evaluated SII, SIRI, PII, or GSH in patients with PTB, investigated these indices as markers for assessing disease severity, progression, or treatment effectiveness, and used standardized measurement methods for these parameters. This study represents a qualitative narrative review of the available literature. The methodological quality of included observational studies was assessed using the Newcastle–Ottawa Scale.

Five authors independently conducted the investigation process, including literature screening, full-text eligibility assessment, and data extraction. Any discrepancies were resolved through consensus discussion, and when necessary, by consultation with the author that supervised the research activity planning and execution.

We aimed to identify studies that evaluated whether SII, SIRI, PII and GSH can be utilized in the detection and predictability of pulmonary TB. Therefore, we conducted a systematic search to gather and synthesize current data on the relationship between these indices and pulmonary TB outcomes. Appendix A Table A1 presents a comprehensive summary of key study characteristics—including parameters (SII, SIRI, PII, and GSH), sample sizes, and main findings—to facilitate cross-study comparison and trend identification. We focused on studies evaluating their sensitivity, specificity, and predictive accuracy for TB diagnosis, disease severity assessment, and treatment response monitoring. Our goal was to critically assess the available evidence to determine if SII, SIRI, PII, and GSH could serve as supportive tools in clinical decision-making for TB patients, ultimately contributing to better management strategies and improved patient outcomes.

The indices were calculated using the following standard formulas: SII = (platelet count × neutrophil count)/lymphocyte count, SIRI = (neutrophil count × monocyte count)/lymphocyte count, PII = (CRP × neutrophil count)/lymphocyte count or equivalently CRP × NLR.

## 3. Results

For clarity, studies included in this review are discussed within two distinct analytical frameworks: diagnostic performance and prognostic value. Diagnostic performance refers to the ability of SII, SIRI, PII, or GSH to discriminate between disease states (e.g., active TB versus latent infection or TB versus non-tuberculous conditions), whereas prognostic value refers to their ability to predict disease severity, microbiological response, or clinical outcomes in patients with established PTB.

### 3.1. Relationship Between Systemic Immune-Inflammation Index and the Prognosis of Pulmonary TB

The systemic immune-inflammation index (SII) has emerged as a promising marker for various respiratory conditions. A Chinese retrospective analysis that included 1327 cases of active PTB showed that SII correlates strongly with inflammatory burden and bacterial load in active TB, serves as an independent diagnostic and prognostic marker, decreases after treatment, and improves specificity when combined with T-SPOT.TB [38]. Higher SII levels are associated with active disease and can help distinguish pulmonary TB from non-tuberculous lung diseases when combined with other markers [38].

Evidence from other pulmonary conditions suggests broader prognostic utility of this index; however, the present section is primarily focused on TB-specific cohorts, with selected non-tuberculous examples provided for contextualization. Along this line of reasoning, elevated SII has been linked to a higher prevalence of chronic obstructive pulmonary disease (COPD) and increased mortality risk in COPD patients [39]. In TB patients with hepatitis B, SII, together with other immune-inflammatory markers, has shown good predictive performance for drug-induced liver injury [40]. Furthermore, SII has demonstrated potential as a biomarker for acute pulmonary embolism (APE), with different cut-off values predicting APE occurrence, severity, and mortality [41]. Collectively, these findings highlight the broader applicability of SII as a marker of inflammatory burden across pulmonary diseases.

In a study by Ștefanescu et al., SII demonstrated a significant decline after two months of intensive-phase therapy, corresponding with sputum culture conversion [42], supporting its potential utility as a prognostic biomarker for treatment success in PTB [42]. In patients with interstitial lung disease and pneumonia, a multicenter retrospective cohort study found that elevated SII levels were associated with adverse clinical outcomes and independently predicted 90-day mortality [43]. Similarly, research on advanced non-small-cell lung cancer (NSCLC) showed that elevated SII correlated with reduced progression-free survival (PFS) and overall survival (OS), functioning as an independent prognostic indicator [44]. Furthermore, a study by Biswas et al. demonstrated that mid-treatment SII measurements obtained during radiotherapy for stage III NSCLC served as significant predictors of both OS and PFS [45]. Collectively, these findings suggest that SII represents a valuable biomarker for monitoring disease progression and evaluating treatment outcomes across diverse respiratory pathologies, including TB and lung cancer. SII is derived from routine complete blood count (CBC) parameters:

SII = (Platelet count × Neutrophil count)/Lymphocyte count

This composite index integrates three critical immunopathological processes: neutrophil-driven inflammation, lymphocyte-mediated immune competence, and platelet involvement in inflammatory signaling [46].

In TB, research has shown that *Mtb* elicits a robust neutrophil response during early and active disease stages [46]. A study investigating the association between peripheral blood inflammation indices and cavitary PTB found that SII levels were significantly elevated in patients with cavitary TB, and higher SII values correlated with anxiety and depression symptoms in TB patients, although SII did not function as an independent risk factor for cavitation [47]. Research by Ștefanescu et al. indicated that concurrent lymphopenia—attributable to immune exhaustion and lymphocyte redistribution to sites of infection—further disrupted immune homeostasis [42]. Additionally, studies have demonstrated that platelets assume active roles in granuloma formation and macrophage modulation [48].

In a five-year cohort study, Ji et al. revealed that SII served as an independent predictor of unfavorable outcomes in PTB, with higher SII levels demonstrating a linear dose–response relationship with adverse risk; the highest quartile (Q4) conferred a 45% increased hazard of poor outcomes compared to the lowest quartile (Q1) [49]. Consequently, research suggests that elevated SII reflects the inflammatory–immune dysregulation characteristic of progressive pulmonary TB [50].

According to the available data, high SII values are associated with active TB versus latent infection [50]; cavitary or multilobar disease [47]; high mycobacterial burden (sputum smear grade 2+/3+); delayed 2-month sputum culture conversion [42]; elevated acute-phase reactants (CRP, ESR, and ferritin) [47]; and TB-related complications, including pleural effusion and respiratory failure [51].

Research indicates that low SII values are observed in latent TB infection (LTBI); early treatment response characterized by declining neutrophil counts and rising lymphocyte counts [42]; radiological improvement with sputum culture conversion; and post-treatment remission.

Although specific cut-off values vary across studies, converging evidence suggests the following thresholds: in a retrospective study that included 1233 patients with PTB, SII ≥ 1252 predicted cavitary TB (AUC 0.628) [47]; some studies indicate that SII > 700–900 suggests extensive disease; SII > 1000 signifies severe inflammation; and SII < 500 suggests milder disease or favorable early treatment response [52]. Research has shown that persistently elevated SII despite appropriate therapy may predict treatment failure or disease relapse [42]. All cut-off values cited in this section are derived from the original studies referenced and were not calculated by the present authors. These thresholds represent study-specific ROC-based or empirically defined values and should not be interpreted as universal clinical decision limits.

Given its accessibility, methodological simplicity, and cost-effectiveness, SII represents a promising tool for risk stratification and treatment monitoring in TB management.

### 3.2. Relationship Between Systemic Inflammation Response Index and the Prognosis of Pulmonary TB

Recent studies have demonstrated the prognostic utility of the systemic inflammation response index (SIRI) across diverse pulmonary pathologies. Findings from non-tuberculous pulmonary diseases indicate that SIRI may have broader prognostic relevance; however, the present section is primarily focused on evidence derived from TB-specific cohorts. In a study examining TB-associated obstructive pulmonary disease (TOPD), researchers found that elevated SIRI values significantly predicted TOPD risk, reflecting the contribution of the monocyte–macrophage axis to chronic inflammatory processes [53]. In patients with lung adenocarcinoma receiving epidermal growth factor receptor tyrosine kinase inhibitors (EGFR-TKIs), Wang et al. demonstrated that low pretreatment SIRI correlated with improved overall survival [54]. In research focusing on smear-negative PTB, investigators showed that SIRI demonstrated auxiliary diagnostic value, although the pan-immune-inflammation index exhibited superior diagnostic accuracy [55,56]. In a study of idiopathic pulmonary arterial hypertension (IPAH), SIRI independently predicted clinical deterioration and correlated with hemodynamic disease severity [55]. SIRI is calculated using the following formula:

SIRI = (Neutrophils × Monocytes)/Lymphocytes

By integrating the pro-inflammatory activity of neutrophils and monocytes with the immunoregulatory function of lymphocytes, SIRI reflects both the intensity of systemic inflammation and the degree of immune suppression—cardinal features of active PTB [53]. These immunopathological processes provide the biological foundation for utilizing SIRI as a disease severity biomarker.

SIRI indicates robust performance as a marker of disease severity and prognosis in PTB. In a study by He et al., SIRI was significantly elevated in patients with cavitary PTB and functioned as an independent risk factor for cavitation, with proposed diagnostic thresholds of approximately 2.095, exhibiting high sensitivity despite limited specificity [47]. In a five-year cohort study, elevated SIRI demonstrated a positive linear association with unfavorable long-term outcomes, underscoring its prognostic value in survival prediction [49]. The independent predictive value of SIRI for cavitary disease was further validated in a retrospective study by Song et al. (*n* = 293 PTB patients), wherein its incorporation into nomogram prediction models revealed its association with amplified systemic inflammatory responses [57].

In a study by Wang et al., SIRI independently predicted severe PTB, demonstrating moderate diagnostic performance (OR 2.742; AUC 0.689) and identifying hyper-inflammatory disease states [58]. Furthermore, research by Chai et al. showed that SIRI exhibited substantial auxiliary diagnostic value for smear-negative PTB, achieving high discriminatory accuracy (AUC 0.82) with significantly elevated levels compared to nontuberculous pulmonary infections [54]. The threshold values reported herein are taken directly from the cited literature and were not independently derived in this review. They reflect study-specific ROC-derived or empirically determined cut-offs and should not be regarded as universally applicable clinical decision criteria.

Relevant findings have established that monocytes and macrophages constitute the primary host cells targeted by *Mtb*, and their accumulation correlates with granuloma formation and bacillary burden [56]. Conversely, research has shown that lymphopenia is prevalent in pulmonary TB and reflects impaired cell-mediated immunity [42]. Consequently, elevated SIRI values reflect the immunological dysregulation that drives TB progression.

Studies have associated elevated SIRI with active pulmonary TB versus latent infection; extensive radiological disease, including cavitary and multilobar involvement; higher sputum smear grades (2+/3+); delayed sputum culture and smear conversion; and elevated systemic inflammatory markers, including CRP, ESR, and ferritin [55].

Research indicates that SIRI demonstrates comparable or marginally superior performance to the neutrophil–lymphocyte ratio (NLR) in predicting TB severity [47] and may be particularly valuable in identifying patients at risk of delayed therapeutic response [54].

During effective anti-TB therapy, studies have shown that SIRI typically decreases as neutrophil and monocyte counts decline and lymphocyte counts recover, reflecting resolution of systemic inflammation [42,48]. Thus, the temporal decline in SIRI represents a reliable surrogate marker of treatment response.

It should be noted that most reported SIRI cut-offs originate from East Asian cohorts, and external validation in other geographic and epidemiological settings remains necessary.

Additional evidence supports that elevated SIRI reflects heightened systemic inflammation and impaired lymphocyte-mediated immunity in PTB [54]. Its predictive capacity appears complementary to that of SII and PII, supporting its integration into multi-parameter inflammatory assessment panels [42,47].

### 3.3. Relationship Between Prognostic Inflammatory Index and the Prognosis of Pulmonary TB

The prognostic inflammatory index (PII) is an integrated inflammatory biomarker that combines acute-phase reactants with hematological parameters to assess systemic inflammation and immune status [59]. It is most commonly calculated using the following formula [54]:

PII = (C-reactive protein × neutrophil count)/lymphocyte count.

Two closely related formulations are reported in the literature: PII = (CRP × neutrophil count)/lymphocyte count and PII = CRP × NLR. Because NLR is defined as neutrophils/lymphocytes, these formulations are mathematically equivalent in most settings. However, inter-study comparability may still be influenced by differences in CRP units, laboratory platforms, and the statistical methods used to derive thresholds [60]. By incorporating C-reactive protein (CRP) with neutrophil and lymphocyte counts, research has shown that PII reflects the combined burden of systemic inflammation and immune suppression [42]—processes central to the pathogenesis and progression of active PTB [47,54].

Based on existing findings, CRP increases markedly in response to *Mtb*-induced inflammation, correlating with bacterial burden and acute-phase response activation [54]. Research indicates that neutrophils contribute both to early antimicrobial defense and to tissue damage in advanced disease [47]. Alongside this, lymphopenia, frequently observed in pulmonary TB, reflects impaired cell-mediated immunity [42]. Consequently, investigations have shown that PII increases when CRP and neutrophil counts rise concurrently with declining lymphocyte counts, thereby accurately reflecting the inflammatory–immune dysregulation characteristic of severe pulmonary TB [51,54].

Published data indicate an association between elevated PII with multiple markers of disease severity and activity, such as active pulmonary TB compared to latent infection [54], extensive radiological disease, including cavitary and multilobar involvement [47], increased bacillary burden and delayed sputum conversion [52], elevated systemic inflammatory markers, including CRP, ESR, and ferritin [54], and TB–HIV co-infection, where research has shown that PII demonstrates substantially elevated levels due to profound immune dysregulation [51].

Chai et al. conducted a retrospective case–control study of 60 bacteria-negative PTB patients and demonstrated that PII showed exceptional diagnostic capability for smear-negative pulmonary TB, with an AUC of 0.84 and specificity of 82.86%—the highest among all inflammatory indices examined—thereby exceeding the diagnostic accuracy of both SII and SIRI [54]. The cut-off points presented in this section are based exclusively on previously published studies and were not generated by the authors. Accordingly, they represent cohort-specific definitions and should be interpreted within the context of the respective study populations rather than as standardized clinical thresholds.

The incorporation of CRP, which amplifies the inflammatory signal, results in PII showing more marked elevations than SII or SIRI in severe disease conditions, as demonstrated in another study of Chai et al. [54].

Findings have shown that PII decreases during effective anti-TB therapy as systemic inflammation subsides [52]. This reduction is primarily driven by declines in CRP and neutrophil counts [54], typically accompanied by radiological improvement [47]. Alongside this, concurrent recovery of lymphocyte counts further reflects immunological restoration and contributes to declining PII values [42]. Studies suggest that progressive reduction in PII is therefore considered a favorable indicator of treatment response [52].

Research indicates that PII demonstrates prognostic performance comparable to SII and SIRI in predicting TB severity [47]. Studies suggest that its integration into multi-parameter inflammatory assessment panels may enhance risk stratification and facilitate early identification of patients at risk for poor treatment outcomes [54].

### 3.4. Relationship Between Glutathione and the Prognosis of Pulmonary TB

Glutathione (GSH) is the most abundant intracellular antioxidant and serves as a central regulator of redox homeostasis, immune function, and antimicrobial defense [60,61]. In the context of *Mtb* infection, research has demonstrated that GSH plays a critical role in macrophage-mediated bacterial killing, cytokine regulation, and protection against oxidative damage [62]. This function is particularly relevant in chronic inflammatory diseases such as PTB, where studies indicate that oxidative stress is markedly elevated [63]. Most studies assessed total and reduced (GSH) glutathione fractions, with reduced GSH representing the biologically active antioxidant form.

Substantial evidence demonstrated that GSH levels are diminished in patients with both pulmonary and extrapulmonary TB, indicative of compromised antioxidant defenses and heightened oxidative stress; in a study by Dalvi et al. and research by Venketaraman et al., this depletion was shown to persist even after six months of treatment in some cases [64,65]. Several studies revealed that although baseline GSH is markedly decreased compared to healthy controls, it progressively increases during anti-TB therapy, recovering toward normal values and correlating positively with zinc and other antioxidant markers such as total antioxidant capacity (TAC) and superoxide dismutase (SOD) [66,67]. Research has shown that progressive normalization of GSH occurs during effective anti-TB therapy [60].

Furthermore, a study by Petrillo et al. identified an inverse association between GSH levels and the degree of disease severity [68]. Studies by Venketaraman et al. and colleagues demonstrate that plasma and intracellular GSH levels are significantly lower in active pulmonary TB patients versus healthy controls [65]. 

Relevant findings suggests that GSH plays a pivotal role in host immunity through multiple mechanisms: enhanced bacterial killing, where research has shown that GSH facilitates phagolysosome fusion in macrophages, thereby improving intracellular killing of *Mtb* [65]; immune cell function, wherein studies demonstrate that GSH depletion impairs T-cell activation and cytokine production (IFN-γ, TNF-α), weakening cell-mediated immunity [69]; oxidative stress mitigation, where investigations reveal that GSH neutralizes reactive oxygen and nitrogen species generated during chronic inflammation, thereby reducing tissue destruction [69]; and cytokine regulation, with research showing that GSH supports antimycobacterial activity and redox homeostasis. Alongside this, studies have found that supplementation with N-acetylcysteine (NAC) enhances intracellular control of *Mtb* while reducing pro-inflammatory cytokine production [65].

Clinical data associates reduced GSH concentrations with more aggressive disease features, including widespread pulmonary involvement [69], elevated bacillary burden and oxidative stress [65], impaired host immune control [63], and delayed conversion with greater clinical morbidity and complication risk [65].

Because available findings demonstrate that GSH reflects both oxidative stress burden and immune capacity, its depletion is considered a negative prognostic indicator [61]. Research conducted by Petrillo et al. revealed that patient-specific differences in GSH levels correspond to individualized oxidative stress responses and metabolic adaptation in TB, highlighting its potential role as a biomarker for disease status and therapeutic efficacy [68].

Emerging observations suggest that adjunctive therapies aimed at restoring GSH levels may improve immune function and reduce oxidative injury in TB patients [65]. However, studies emphasize that large-scale randomized controlled trials are required to confirm these preliminary findings and establish optimal dosing regimens [63].

A practical distinction is that, unlike SII, SIRI, and PII (derived from routine CBC and CRP), GSH measurement typically requires specialized assays and standardized sample handling, which may limit availability in many high-burden, resource-constrained settings. Nevertheless, GSH remains biologically informative and may be particularly valuable in research contexts, mechanistic studies, and the evaluation of adjunctive host-directed therapeutic strategies.

## 4. Discussion

PTB involves complex immunopathological mechanisms including chronic inflammation, immune dysregulation, and oxidative stress [1,2,3]. This review demonstrates that hematological inflammatory indices—SII, SIRI, and PII—alongside GSH, represent promising prognostic biomarkers reflecting these interconnected pathways. Our analysis of 12 studies reveals compelling evidence for their clinical utility in TB diagnosis, disease severity assessment, and treatment monitoring.

Reported cut-offs for SII, SIRI, and PII vary across studies. This heterogeneity likely reflects differences in study populations (e.g., comorbidity burden, HIV co-infection, nutritional status), TB phenotypes and severity definitions (e.g., cavitary disease, smear grade, extent of radiologic involvement), endpoints (diagnostic discrimination vs. prognostic outcomes vs. early treatment response), laboratory methodologies and platforms, and statistical strategies used for threshold selection (e.g., ROC-derived optimal cut-offs versus quantile-based categorization). Accordingly, the reported thresholds should be interpreted as study-specific ranges rather than universal clinical decision limits.

Because SII, SIRI, and PII share common hematological components—particularly neutrophil and lymphocyte counts—these indices are mathematically interrelated and may exhibit substantial collinearity when incorporated into multivariable models. While the present review does not perform original statistical modeling, future studies evaluating combined predictive panels should formally assess multicollinearity using correlation matrices, variance inflation factors, or dimensionality-reduction techniques.

It is important to distinguish between diagnostic and prognostic applications of inflammatory indices in tuberculosis. Several studies evaluated biomarker performance in already diagnosed cohorts, thereby addressing prognostic rather than diagnostic utility. In this review, we therefore differentiate diagnostic discrimination from prognostic prediction to avoid conceptual overlap and to ensure accurate interpretation of reported performance metrics.

SII is the most extensively studied marker in TB. In the study by Ștefanescu et al., SII demonstrated significant decline in patients achieving sputum culture conversion, supporting its utility as a prognostic biomarker [42]. Evidence indicates SII correlates strongly with inflammatory burden and bacterial load, improving specificity when combined with T-SPOT.TB [38].

Research has shown that *Mtb* elicits robust neutrophil responses during active disease [46], while concurrent lymphopenia disrupts immune homeostasis [42]. Studies demonstrate that platelets assume active roles in granuloma formation [48]. In a five-year cohort study, Ji et al. revealed that SII served as an independent predictor of unfavorable outcomes, with the highest quartile conferring 45% increased hazard [49]. Studies consistently show that high SII values are associated with active TB, cavitary disease [47], high mycobacterial burden, delayed sputum conversion [42], elevated acute-phase reactants [47], and TB-related complications [51,52].

Persistently elevated SII despite therapy may predict treatment failure [42]. Beyond TB, elevated SII independently predicted 90-day mortality in interstitial lung disease [43] and reduced survival in advanced NSCLC [44].

SIRI provides complementary information by incorporating monocyte counts, highlighting the critical monocyte–macrophage axis in TB pathogenesis. Studies establish that monocytes and macrophages constitute primary host cells targeted by *Mtb*, with accumulation correlating with granuloma formation and bacillary burden [56].

In a study by He et al., SIRI was significantly elevated in cavitary PTB and functioned as an independent risk factor for cavitation (threshold approximately 2.095) [47]. Song et al. validated SIRI’s predictive value in nomogram models (*n* = 293) [57]. Wang et al. demonstrated SIRI independently predicted severe PTB (OR 2.742; AUC 0.689) [58], while Chai et al. showed substantial diagnostic value for smear-negative PTB (AUC 0.82) [54].

Documented findings associate elevated SIRI with active TB, extensive radiological disease, higher sputum smear grades, delayed conversion, and elevated inflammatory markers [55]. During effective therapy, SIRI typically decreases as neutrophil and monocyte counts decline and lymphocyte counts recover [42].

PII distinguishes itself by integrating CRP with neutrophil and lymphocyte counts, demonstrating particular sensitivity in severe TB and TB–HIV co-infection. Studies show CRP increases markedly in response to *Mtb*-induced inflammation [54]. PII increases when CRP and neutrophil counts rise with declining lymphocyte counts, accurately reflecting inflammatory–immune dysregulation in severe TB [51].

Chai et al. demonstrated that PII showed increased diagnostic capability for smear-negative PTB, with AUC of 0.84 and specificity of 82.86%—exceeding both SII and SIRI [54]. The superior diagnostic performance of PII may be explained by the incorporation of CRP, which reflects acute-phase inflammatory signaling and bacterial burden, thereby adding a dynamic inflammatory dimension not captured by cell-count-based indices alone. CRP incorporation results in PII showing more marked elevations than SII or SIRI in severe disease [54]. Studies associate elevated PII with active TB [59], extensive radiological disease [47], increased bacillary burden [52], elevated inflammatory markers [54], and TB–HIV co-infection with substantially elevated levels [51].

GSH provides mechanistic insight by reflecting oxidative stress burden and immune capacity—dimensions not captured by cell count-based indices. Research demonstrates GSH plays critical roles in macrophage-mediated bacterial killing, cytokine regulation, and protection against oxidative damage [62].

The proposed integration of GSH (expressed in concentration units) with dimensionless hematological indices should be interpreted as a conceptual and biological framework rather than a statistically derived composite score. Any future multivariate predictive modeling would require appropriate standardization or normalization procedures and could benefit from dimensionality-reduction approaches such as principal component analysis.

Existing data demonstrates that GSH levels are diminished in pulmonary and extrapulmonary TB. In studies by Dalvi et al. and Venketaraman et al., this depletion persisted after six months of treatment in some cases [64,65]. Studies demonstrate plasma and intracellular GSH levels are significantly lower in active PTB versus healthy controls [65]. Petrillo et al. identified an inverse association between GSH levels and disease severity [68].

The findings from several studies indicated baseline GSH progressively increases during anti-TB therapy, recovering toward normal values and correlating positively with zinc and other antioxidant markers [66,67]. GSH’s mechanistic roles include the following: enhanced bacterial killing through phagolysosome fusion [65]; immune cell function, wherein GSH depletion impairs T-cell activation and cytokine production [69]; oxidative stress mitigation [69]; and cytokine regulation. Studies found N-acetylcysteine supplementation enhances intracellular *Mtb* control while reducing pro-inflammatory cytokine production [65].

Additionally, low GSH levels have been linked to extensive pulmonary involvement [69], higher mycobacterial burden with increased oxidative stress [65], impaired immune responses [63], and a greater risk of delayed conversion and complications [65]. Petrillo et al. revealed that patient-specific GSH differences correspond to individualized oxidative stress responses, highlighting its potential as a biomarker for disease status and therapeutic efficacy [68].

Common tuberculosis comorbidities may act as important confounders of inflammatory and oxidative stress biomarkers. HIV infection is associated with lymphopenia and chronic immune activation, while diabetes mellitus is characterized by systemic inflammation and increased oxidative stress, both of which may independently influence SII, SIRI, PII, and GSH values. These conditions should therefore be accounted for in future studies evaluating the clinical utility of these biomarkers.

Preliminary evidence suggests that patients with drug-resistant TB may exhibit higher baseline inflammatory indices and more pronounced oxidative stress, reflecting prolonged disease activity and delayed microbiological response. However, dedicated studies in MDR-TB populations remain limited and warrant further investigation.

SII, SIRI, PII, and GSH are complementary tools capturing different TB immunopathology facets. Research indicates that integrating these biomarkers into multi-parameter panels may enhance risk stratification [42,47,54]. Studies consistently show effective therapy is accompanied by declining SII, SIRI, and PII alongside rising GSH [42,52,60,66,67].

Importantly, although we propose an integrated framework combining SII, SIRI, PII, and GSH to capture complementary dimensions of TB immunopathology, the current evidence base largely evaluates these biomarkers separately. Direct head-to-head comparisons and multivariable models incorporating these markers within the same cohorts remain limited. Therefore, the ‘integrated approach’ should be interpreted as a conceptual synthesis that requires prospective validation in standardized, multicenter studies before routine clinical implementation.

These biomarkers offer practical advantages for clinical implementation. All markers derive from routine laboratory tests—complete blood counts and CRP—that are widely available and inexpensive. Inflammatory indices derived from blood counts and CRP may be influenced by conditions independent of TB activity, including acute or chronic co-infections, metabolic comorbidities (e.g., diabetes), liver disease, smoking status, anemia and other hematologic disorders, nutritional deficiencies, pregnancy, and concomitant medications (e.g., corticosteroids or immunomodulatory therapies). These potential confounders should be considered when interpreting SII, SIRI, and PII and should be systematically adjusted in future prospective cohorts.

## 5. Conclusions

SII, SIRI, PII, and GSH represent accessible, cost-effective, and biologically relevant biomarkers with substantial potential for prognostic assessment in PTB. Elevated values of SII, SIRI, and PII predict active and severe disease, increased bacillary burden, and delayed microbiological response, whereas declining values reflect favorable treatment outcomes. Conversely, reduced GSH levels identify patients with heightened oxidative stress, impaired immunity, and adverse clinical trajectories.

This review demonstrates that hematological inflammatory indices (SII, SIRI, PII) and GSH represent promising prognostic biomarkers in PTB. SII emerges as the most extensively validated marker. SIRI provides complementary information incorporating the monocyte–macrophage axis. PII demonstrates superior diagnostic accuracy in smear-negative TB. GSH uniquely reflects oxidative stress burden and immune capacity.

The evidence supports clinical utility for early diagnosis, risk stratification, treatment monitoring, and prognostic assessment. Their derivation from routine laboratory tests makes them particularly suitable for resource-limited, high-burden settings.

However, significant knowledge gaps constrain immediate implementation. To advance the field, standardized multicenter prospective studies are urgently needed to validate optimal cut-offs, evaluate multi-parameter panels, and determine cost-effectiveness. Mechanistic research may identify therapeutic targets, while interventional trials could establish causal relationships. Additionally, the incremental prognostic value of these biomarkers beyond conventional clinical, radiological, and microbiological parameters must be rigorously established.

Integrating SII, SIRI, PII, and GSH into TB management holds substantial promise for improving diagnostic accuracy, treatment monitoring, and patient outcomes. Addressing identified research gaps through rigorous investigations will be essential to realize this promise and will contribute to global TB control efforts.

In clinical practice, these biomarkers—particularly SII, SIRI, and PII—may be most useful as adjuncts to standard monitoring (sputum microscopy/culture or molecular testing, clinical assessment, and radiological evaluation), supporting earlier risk stratification and identification of patients who may benefit from closer follow-up rather than replacing established diagnostic or treatment response tools. Future clinical trials should incorporate standardized, longitudinal measurement of SII, SIRI, PII, and GSH at predefined treatment milestones, evaluate their incremental prognostic value beyond conventional microbiological and radiological monitoring, and assess their role in guiding risk-adapted therapeutic strategies.

## Data Availability

All data relevant to the review are included within the article. No new data sets were generated.

## Appendix A

**Table A1:** Study characteristics, SII, SIRI, PII and GSH parameters, and key findings from empirical investigations in PTB patients.

Author (Year)	Study Design	Sample Size (*n*)	Population	Measurement Methods	Parameters	Endpoints	Key Findings
Song et al. 2024 [57]	Retrospective, observational, single-site, randomized allocation to training and validation sets	293	293 PTB patients	Chest computed tomography (CT) for identifying cavitation Extraction of demographic characteristics, clinical symptoms, and laboratory parameters from medical records Calculation of SIRI using laboratory parameters Statistical analyses: chi-square tests, Fisher’s exact tests, Mann–Whitney U-tests	SIRI	The primary endpoint is the construction and validation of a nomogram to identify the risk of cavitation in PTB patients based on clinical metrics such as sputum smear positivity, smoking history, HbA1c, hemoglobin (HB), and SIRI.	Elevated SIRI levels indicate a heightened inflammatory state, significantly associated with cavitation. This finding aligns with research in other inflammatory conditions, where SIRI has been shown to predict outcomes. The novel association between SIRI and cavitary PTB underscores the importance of systemic inflammation in the pathogenesis of cavitary lesions in PTB.
Wang et al. 2025 [58]	Retrospective, observational study conducted at a single institution.	182	Non-severe PTB: 112 patients Severe PTB: 70 patients	Laboratory tests: WBC, neutrophil, lymphocyte, monocyte, and platelet counts; VEGF, IL-6, and CRP levels. Calculation of inflammatory indices: NLR, MLR, PLR, SII, SIRI. Statistical analysis using SPSS version 25.0 and R version 4.3.3. Statistical tests: Kolmogorov–Smirnov test, independent-samples t-test, Mann–Whitney U test, chi-squared test, Fisher’s exact test. ROC curve analysis for predictive performance evaluation.	SII, SIRI	Area under the ROC curve (AUC): 0.859 Sensitivity: 80.00% Specificity: 83.04% Youden index: 0.630	SIRI is a significant predictor of severe PTB.
Ji et al. 2025 [49]	Observational study Retrospective study Single-site study Stratified design	4027	4027 PTB patients	Blood tests from the laboratory information system were used to calculate inflammatory indices. Clinical data collection at the Shenzhen Third People’s Hospital. Statistical analyses: Cox regression, ROC analyses, Kaplan–Meier estimates, Log-rank tests.	SII, SIRI	Successful treatment (cured and treatment completed) Unfavorable outcome (treatment failure and death) Survival time (interval between treatment initiation and outcome) Predictive accuracy of inflammatory indices for treatment outcomes	Elevated levels of SII and SIRI are associated with an increased risk of unfavorable outcomes in TB patients. The combination of these indices improves the predictive accuracy of TB treatment outcomes.
He et al. 2024 [47]	Retrospective, observational, controlled, single-site	1233	Case group: 518 patients with cavitary PTB Control group: 715 patients with non-cavitary PTB	Not mentioned (the paper does not specify the measurement methods for inflammation indices)	SII, SIRI	When cavitary PTB was taken as the endpoint, the optimal diagnostic threshold of CRP was 35.365 (area under the ROC curve (AUC) = 0.601), NLR was 5.740 (AUC = 0.595), MLR was 0.525 (AUC = 0.577), PLR was 198.255 (AUC = 0.602), SII was 1252.045 (AUC = 0.628), and SIRI was 2.095 (AUC = 0.605), respectively.	CRP, NLR, MLR, PLR, SII, and SIRI levels were significantly higher in cavitary PTB patients. Gender, CRP, PLR, and SIRI were independent risk factors for cavitary PTB. The combination of CRP, PLR, and SIRI showed higher sensitivity for cavitary diagnosis. These markers are promising for screening and ruling out PTB with cavitary.
Yu et al. 2024 [38]	Retrospective, observational, single-center study	2030	Active PTB cases: 1327 Non-tuberculous lung diseases cases: 703	T-SPOT.TB test: Peripheral venous blood collection, isolation and counting of PBMCs, incubation with antigens (ESAT-6 and CFP-10), spot counting, and result interpretation. Blood collection: Overnight fast, venipuncture with EDTA-K2, sodium citrate, and gel progel anticoagulants. Blood analysis: Standard operating procedures on LBY-XC40B, AU5400, ACLTOP700, and SYSMEX XE-2100 blood analyzers for ESR, CRP, blood coagulation indexes, and blood cell composition.	SII	Sensitivity: 52.6%, 53.0%, 55.8%, 44.7% Specificity: 82.5%, 83.2%, 95.8%, 80.1% Positive Predictive Values: 85.0%, 85.6%, 84.1%, 89.6% Optimal diagnostic thresholds: ESAT-6 (21.50 SFCs/10^6 PBMC), CFP-10 (22.50 SFCs/10^6 PBMC), SII (2128.32), Fibrinogen (5.02 g/L)	Higher values of ESAT-6, CFP-10, SII, and Fibrinogen in active PTB compared to non-tuberculous lung diseases. SII and Fibrinogen are positively correlated with CRP but not with ESAT-6 and CFP-10. Established optimal diagnostic thresholds for ESAT-6, CFP-10, SII, and Fibrinogen. These markers are independent risk factors for active PTB. Combination of markers shows varying specificity, sensitivity, and positive predictive values. SII and Fibrinogen are positively correlated with TB inflammation and bacterial load.
Ştefanescu et al. 2021 [42]	Cohort study Observational study Multi-site study	90	90 newly diagnosed PTB patients	Hematological measurements: Abacus 5 Analyzer with 5-part diff (Diatron, Germany) Quality control: Twice-daily high, normal, and low internal quality control (IQC); external quality controls (EQS); annual quality assurance Laboratory accreditation: Romanian National Accreditation System (Romanian Accreditation Association-RENAR) in accordance with international standard ISO 15189/2013 [70] ESR measurement: Westergren method	SII	Culture conversion (from positive to negative) after the 2-month intensive phase of treatment is the primary endpoint. The study mentions that in 63 out of 90 patients, there was a significant decrease in several hematological parameters after treatment, indicating culture conversion as a key endpoint.	Significant decrease in blood cell count, hemoglobin, plateletcrit, erythrocyte sedimentation rate, MLR, NLR, PLR, and SII in patients who turned culture negative after treatment.
Chai et al. 2023 [54]	Retrospective case–control study, controlled, single-site	130	Case group: 60 patients with bacteria-negative PTB Control group: 70 patients with nontuberculous pulmonary infection	Collection of baseline data and laboratory data Detection method: Fasting venous blood samples, immunoturbidimetry Data analysis: SPSS 27.0, MedCalc 20.1	PII, SIRI	The results showed that area under curve (AUC) value, sensitivity and specificity were, respectively, 0.84 [95%CI (0.77, 0.90)], 78.33% [95%CI (65.8, 87.9)] and 82.86% [95%CI (72.0, 90.8)] of PII; 0.82 [95%CI (0.74, 0.88)], 80.00% [95%CI (67.7, 89.2)] and 77.14% [95%CI (65.6, 86.3)] of SIRI; 0.81, 73.33% and 75.71% of NLR; 0.82, 86.67% and 65.71% of NLR + PII; 0.83, 80.00% and 75.71% of NLR + SIRI; 0.82,78.33% and 78.57% of PII + SIRI; 0.83, 78.33% and 78.57% of NLR + PII + SIRI; and the AUC value and specificity of PII were the highest, while the sensitivity of NLR + PII had the maximum value.	PII had the highest AUC value and specificity in diagnosing bacteria-negative pulmonary TB. NLR + PII had the highest sensitivity in diagnosing bacteria-negative pulmonary TB. PII and SIRI have clinical application value in differentiating between nontuberculous and tuberculous pulmonary infections. These indices can aid in reducing diagnostic time and cost, potentially improving patient outcomes
Venketaraman et al. 2008 [65]	Pilot study Small-scale study with 12 subjects (6 healthy controls, 6 active TB patients) Controlled study (comparing healthy controls with active TB patients) Single-site study (conducted at the Lattimore Clinic)	12	Active TB patients: 6 (without HIV infection)	GSH levels: Spectrophotometry using a glutathione assay kit (Calbiochem) Protein estimation: Bradford protein microassay (Bio-Rad) Cytokine levels: Liqui-Chip cytokine detection kit (Qiagen) and Beadlyte kit Intracellular viability of H37Rv: Plating on 7H11 agar	GSH	Extent of decrease in GSH levels in patients with active TB Relationship between GSH levels and ability to kill intracellular M. tuberculosis Effect of NAC treatment on GSH levels and control of intracellular M. tuberculosis infection Modulation of cytokine levels (IL-10, IL-6, TNF-a, IL-1) in response to NAC treatment	GSH levels are significantly reduced in peripheral blood mononuclear cells (PBMC) and red blood cells (RBC) isolated from TB patients. GSH plays a crucial role in controlling mycobacterial replication and modulating immune responses. GSH modulates cytokine synthesis and promotes host immune responses.
Meca et al. 2021 [67]	Pilot study Prospective study Before-and-after design Observational study	30	30 newly diagnosed PTB	Spectrophotometric methods for TBARS, SOD, CAT, GPx, TAC, and GSH. Ransod kit for SOD activity. Ransel kit for GPx activity. Aebi method for CAT activity. Ellman’s reagent for GSH concentration. UV/VIS spectrophotometers for absorbance measurements.	GSH	TBARS (thiobarbituric acid reactive species) TAC (total antioxidant capacity) SOD (superoxide dismutase) GPx (glutathione peroxidase) CAT (catalase) GSH (reduced glutathione)	Increased values of GSH, CAT, and SOD were noted at T2 compared to T0, while GPx, TAC, and thiobarbituric acid reactive species decreased. Monitoring oxidative stress and antioxidant status could improve TB management. Increased oxidative species and reduced antioxidant status are involved in TB pathogenesis.
Qi et al. 2020 [66]	Case–control study; Non-randomized; Longitudinal; Controlled (with healthy controls)	198	PTB patients: 86 individuals Healthy controls: 112 individuals	Liver enzymes AST and ALT: Clinical chemistry automated analyzer (Beckman Coulter, DXC800) Oxidative stress markers: - MDA: TBA method -GPx: Colorimetric method - GSH: Spectrophotometric method - SOD: WST-1 method - CAT: Visible light Trace elements: Inductively coupled plasma mass spectrometry (ICP-MS; NexlON 350, PerkinElmer)	GSH	Oxidative stress (OS) markers: SOD, CAT, GSH, MDA Trace element levels: Zn, Cu, Se	MDA: Increased before treatment, decreased during treatment GSH: Decreased before treatment, increased during treatment CAT: Decreased before treatment, increased during treatment SOD: Decreased before treatment, increased during treatment After 6 months of anti-TB treatment, antioxidant levels returned to normal, reducing oxidative stress. Trace elements (Zn, Se, Cu) were initially lower in TB patients but increased during treatment.
Petrillo et al. 2022 [68]	Observational study, controlled, single-site	48	Healthcare workers (Ctrls): 40 Active TB patients: 8	GSH levels: Enzymatic re-cycling assay using Thi-oStar glutathione detection reagent and EnSpire Multimode Plate Reader. NRF2 and gene expression: qRT-PCR using ABI PRISM 7500 Sequence Detection System. Interferon-gamma: Interferon-gamma Release Assay (IGRA) with Quantiferon-TB Gold. Statistical analysis: GRAPHPAD/Prism 5.0 Software and Student’s *t*-test.	GSH	GSH levels in blood NRF2 expression levels Expression levels of NRF2 target genes (SOD1/2, HO-1, GCL)	Measured GSH levels in blood of patients with active TB to understand individual Nuclear factor Erythroid 2-related Factor 2 (NRF2)-mediated redox profiles. Found variability in GSH levels among patients and compared to controls, indicating personalized consumption and synthesis. NRF2 expression showed individual responses, peaking early in therapy and decreasing with longer treatments. NRF2 target genes showed slow activation throughout therapy, varying among patients. Suggests potential for personalized medicine approaches, including tailored antioxidant therapies. Personalized redox signatures and systemic GSH determination could aid in monitoring infection and treatment.
Dalvi et al. 2012 [64]	Stratified design based on disease categories Controlled study with healthy human volunteers as controls Longitudinal/observational study design	235	100 PTB patients 35 Extra-PTB patients 100 subjects Normal control	Collection of venous blood samples in vacutainers Centrifugation to separate serum Analysis of protein carbonyl content Measurement of glutathione concentration using a Randox kit Use of UV-spectrophotometer and auto analyzer for biochemical measurements Statistical analysis using Student’s *t* test	GSH	Increase in serum protein carbonyl levels Decrease in blood glutathione, glutathione peroxidase, and glutathione reductase levels Reversal of these changes after 6 months of antitubercular treatment	Activities of glutathione, glutathione peroxidase, and glutathione reductase were significantly decreased in pulmonary and extra-PTB patients. Antitubercular treatment partially reversed these changes but did not completely restore normal levels.

PTB = pulmonary tuberculosis; GSH = glutathione; NRF2 = Nuclear factor Erythroid 2-related Factor 2; PBMC = peripheral blood mononuclear cells; RBC = red blood cells.
